# Circulating Rhythmic Metabolites and Causal Risk of Type 2 Diabetes in Adults in the Canadian Longitudinal Study on Aging

**DOI:** 10.1111/dom.70616

**Published:** 2026-03-08

**Authors:** Divya Joshi, Talha Rafiq, Marie Pigeyre, Renee de Mutsert, Femke Rutters, David J.T. Campbell, Jean‐Pierre Despres, Andre C. Carpentier, Joris Hoeks, Patrick Schrauwen, Parminder Raina

**Affiliations:** ^1^ Department of Health Research Methods, Evidence, and Impact McMaster University Hamilton Ontario Canada; ^2^ Department of Medicine McMaster University Hamilton Ontario Canada; ^3^ Department of Clinical Epidemiology Leiden University Medical Center Leiden the Netherlands; ^4^ Department of Epidemiology and Data Science Amsterdam University Medical Centre, Location Vrije Universiteit Amsterdam Amsterdam North Holland the Netherlands; ^5^ Cumming School of Medicine, Department of Medicine, Department of Community Health Sciences, Department of Cardiac Sciences University of Calgary Calgary Alberta Canada; ^6^ Department of Kinesiology Laval University Laval Quebec Canada; ^7^ Centre de Recherche du Centre Hospitalier Universitaire de Sherbrooke, Université de Sherbrooke Sherbrooke Quebec Canada; ^8^ Department of Nutrition and Movement Sciences NUTRIM Institute of Nutrition and Translational Research in Metabolism, Maastricht University Maastricht Limburg the Netherlands; ^9^ Institute for Clinical Diabetology, German Diabetes Center, Leibniz Institute for Diabetes Research at Heinrich Heine University Düsseldorf Germany; ^10^ McMaster Institute for Research on Aging, McMaster University Hamilton Ontario Canada; ^11^ Labarge Centre for Mobility in Aging, McMaster University Hamilton Ontario Canada

**Keywords:** circadian rhythm, CLSA, Mendelian randomization, metabolomics, rhythmic, Type 2 diabetes

## Abstract

**Aims:**

Circadian regulation of metabolism is an important factor in metabolic health, yet the role of rhythmic metabolites in Type 2 diabetes development remains poorly understood. This study investigated associations between circulating rhythmic metabolites and incident Type 2 diabetes risk and evaluated causal relationships using two‐sample Mendelian randomisation.

**Materials and Methods:**

We analysed longitudinal data from 9992 community‐dwelling adults aged 45–85 years (49.1% male) in the Canadian Longitudinal Study on Aging with baseline (2012–2015) serum metabolomics data. Untargeted metabolomics profiling was conducted using ultrahigh‐performance liquid chromatography–tandem mass spectrometry. Incident Type 2 diabetes at 3‐year follow‐up was assessed based on diabetes medication use and HbA1c level. Associations between rhythmic metabolites and diabetes risk were evaluated using multivariable binomial regression. Pathway and network analyses were conducted to explore underlying biological mechanisms. Causality was assessed using two‐sample Mendelian randomisation for rhythmic metabolites significantly associated with diabetes risk.

**Results:**

Altogether, 20 rhythmic metabolites were associated with Type 2 diabetes risk, including a subset overlapping with genetic predisposition to chronotype, suggesting potential circadian regulation. Key pathways included leucine, isoleucine and valine biosynthesis and degradation, and glycine, serine and threonine metabolism. Mendelian randomisation analyses revealed causal associations between higher levels of mannose, valine, isoleucine, threonine and sphingomyelin (d18:0/18:0, d19:0/17:0) and higher Type 2 diabetes risk, whereas creatine, glycine, 1‐linoleoyl‐GPC (18:2), 1‐palmitoyl‐2‐oleoyl‐GPE, 1‐palmitoyl‐2‐linoleoyl‐GPE (16:0/18:2) and 1‐stearoyl‐2‐oleoyl‐GPE (18:0/18:1) were protective.

**Conclusions:**

Disruptions in rhythmic metabolites are implicated in Type 2 diabetes pathophysiology through specific metabolic pathways, highlighting the potential for biomarkers to support circadian‐based prevention strategies.

## 1 Introduction

Circadian rhythms play an important role in regulating metabolic homeostasis, by coordinating energy utilisation and storage with daily feeding‐fasting cycles and the sleep–wake cycle [1]. The central circadian clock, located in the suprachiasmatic nucleus of the hypothalamus, synchronises peripheral tissue clocks to regulate hepatic glucose production, pancreatic beta cell insulin secretion, lipid oxidation, and mitochondrial function in metabolic active tissues [1, 2]. Disruption of the circadian rhythm is associated with metabolic dysfunction and increased risk of Type 2 diabetes [3, 4]. Most studies investigating mechanisms underlying Type 2 diabetes have focused on circadian gene expression, with few studies examining metabolites that exhibit rhythmicity [5, 6]. A substantial proportion of the human serum metabolome oscillates over a 24‐h period [7], and emerging evidence suggests that these oscillations are regulated by the endogenous circadian clocks, independent of external factors such as feeding, activity, and sleep [8].

Despite increasing interest in chronometabolism, the role of rhythmic metabolites in cardiometabolic disease risk, particularly Type 2 diabetes, has not been thoroughly investigated. Existing studies are limited by cross‐sectional designs, small sample sizes, controlled experimental settings, targeted metabolomics approaches and lack of causal inference [5, 9]. To address these gaps, we examined associations between previously identified rhythmic metabolites and incidence Type 2 diabetes risk in a large, population‐based cohort. To strengthen causal inference, we complemented our observational analyses with two‐sample Mendelian randomisation (MR). Additionally, we explored whether a subset of these rhythmic metabolites overlapped with genetic predisposition to chronotype, which may provide insight into circadian regulation among metabolites associated with diabetes risk. By integrating chronobiology with metabolomics, this study aims to identify metabolic signatures of circadian dysregulation that may contribute to Type 2 diabetes pathophysiology and inform future prevention strategies.

## 2 Materials and Methods

### 2.1 Study Design and Participants

The Canadian Longitudinal Study on Aging (CLSA) is a longitudinal cohort study involving a nationally generalizable, stratified random sample of 51 338 community‐dwelling individuals aged 45–85 years at baseline (2011–2015) [10, 11]. The CLSA consists of two cohorts: Tracking (*n* = 21 241) and Comprehensive (*n* = 30 097). Participants in the Comprehensive Cohort attend a Data Collection Site for more detailed physical assessments and to provide biological samples. Of the 30 097 participants, blood and urine samples were available for 23 492 participants, from which a random sample of 10 000 participants were selected for metabolomics analyses. The current study utilised baseline and 3‐year follow‐up data (95% retention rate) from participants for whom metabolomics data are available (*n* = 9992). This study was approved by the Hamilton Integrated Research Ethics Board (Project ID: 14106).

### 2.2 Measures

#### 2.2.1 Type 2 Diabetes

Type 2 diabetes status was assessed at baseline and at 3‐year follow‐up using a validated disease ascertainment algorithm [12]. Participants were classified as having diabetes if they indicated diabetes medication use or had an HbA1c level ≥ 6.5%. Diabetes medication status was determined based on drug identification numbers, Anatomical Therapeutic Chemical code starting with A10, drug names, and reason for taking the medication. Participants who did not meet the criteria for Type 2 diabetes were classified as not having Type 2 diabetes. Participants with Type 2 diabetes at baseline were excluded, and incident Type 2 diabetes cases were included as outcomes.

#### 2.2.2 Metabolomics

An untargeted metabolomics analysis of plasma samples at baseline was performed by Metabolon Inc. (Durham, NC, USA). The Ultrahigh Performance Liquid Chromatography‐Tandem Mass Spectroscopy (UPLC‐MS/MS) platform was used for identification and relative quantification of metabolites (see Supporting Information: Methods section titled ‘Metabolomics’) [13]. Metabolites with > 50% of missing values were excluded from the analysis. The missing values for the remaining metabolites were imputed using multiple chained equations using the R package ‘mice’ with five imputations applying random forest as the imputation model. All metabolites were natural log‐transformed, trimmed to remove outliers > 3 standard deviations from the mean, and standardised to a mean of 0 and standard deviation of 1. As plasma samples were obtained at a single time point, metabolite rhythmicity could not be evaluated within the CLSA. Accordingly, metabolites were classified as rhythmic based on consistent evidence of 24‐h oscillation reported in at least three independent studies. A prior review of all published studies to date identified 139 metabolites that have shown 24‐h rhythmicity [7]. Of these, data for 98 metabolites (70.5%) were available in the CLSA and included in downstream analyses (Table S1).

#### 2.2.3 Polygenic Risk Score (PRS) for Chronotype

The UK Biobank is a large population‐based cohort of over 500 000 adults aged 40–69 years at recruitment, enrolled across the United Kingdom between 2006 and 2010, with extensive phenotypic and genetic data. Among the Comprehensive subset, 26 622 participants (93% of European background) were genotyped using the UK Biobank Array [14]. The quality control steps have been detailed elsewhere (see Supporting Information: Methods section titled ‘Polygenic risk score for chronotype’) [15]. PRS were constructed from the summary statistics of genome‐wide association studies (GWAS) conducted in 449 734 individuals of European‐ancestry from the UK Biobank, in order to assess genetic predisposition to two available chronotype indicators: morningness (‘Are you naturally a night person or a morning person?’) and morning person (‘Do you consider yourself to be: definitely a morning person…definitely an evening person?’) [16]. For each indicator, two PRS were generated adjusted for age, sex, study centre, genotyping array/release and population structure (genetic relatedness) (see Table S2), one based on all risk alleles, and one based on risk alleles in core circadian genes (*CLOCK*, *BMAL1*, *PER1*, *PER2*, *PER3*, *CRY1*, *CRY2*, *MTNR1B*), resulting in a total of four scores.

### 2.3 Statistical Analysis

All statistical analyses were two‐tailed, with a statistical significance of 0.05 unless otherwise specified and performed using R Studio.

#### 2.3.1 Association Between Chronotype PRS and Rhythmic Metabolites

To examine whether genetic predisposition to evening chronotype is associated with identified metabolites, a multivariable linear regression was performed between chronotype PRS and individual metabolites, adjusting for covariates identified a priori in the literature, including age, sex, education, smoking, physical activity, diet quality (Prospective Urban Rural Epidemiological (PURE) healthy diet score based on intake of fruits, vegetables, legumes, nuts, fish and dairy) [17], alcohol consumption, BMI, participation in shift work, number of chronic conditions, use of lipid modifying medication and 20 genetic ancestry principal components. Table S3 describes operationalisation of covariates in the analysis. A false discovery rate (FDR) corrected *p* value < 0.05 was considered statistically significant.

#### 2.3.2 Association Between Rhythmic Metabolites and Type 2 Diabetes

A binomial generalised multivariable linear model with a log link function was used to examine the association between rhythmic metabolites and Type 2 diabetes risk, adjusting for covariates including age, sex, education, smoking, adequate physical activity, diet quality, alcohol consumption, BMI, number of chronic conditions and use of lipid modifying medication. As sensitivity analysis, we also adjusted the model for timing of participant visit and fasting status to account for potential diurnal variation in metabolite levels, which produced similar results (data not shown). We performed 1000‐fold bootstrapping for discovery and replication. Each bootstrap randomly split 70% of the data as the discovery set and 30% as the replication set to evaluate model performance. The adjusted relative risk reflects Type 2 diabetes risk associated with a 25% increase in a certain metabolite score, on average across individuals in the study population.

#### 2.3.3 Metabolic Pathway Analysis

Pathway and network analyses were performed using the Kyoto Encyclopedia of Genes and Genomes (KEGG) pathway via MetaboAnalyst 6.0 (http://www.metaboanalyst.ca/) to identify metabolic pathways enriched among rhythmic metabolites that were significantly associated with Type 2 diabetes risk. Enrichment analysis was used to identify the group of metabolites in each functional node using an FDR‐adjusted *p* value of < 0.05.

#### 2.3.4 MR

A two‐sample MR was performed using the ‘MendelianRandomization’ R package to estimate the causal effect of each metabolite that showed a statistically significant association with Type 2 diabetes in the observational analyses. MR instruments were available for 16 of the 20 associated metabolites, based on genome‐wide significant variants (*p* < 5.0 × 10^−8^) identified in two published metabolomics GWAS datasets [18, 19]. Linkage disequilibrium was used to define independent variants according to the 1000 genomes data with an *r* ^2^ threshold of 0.01 and clump distance of 1000 kb implemented in PLINK. Genetic instruments with an *F*‐statistic of < 10 were considered weak instruments and were excluded. MR pleiotropy test function was employed to ensure that the results are free of horizontal pleiotropy. The summary level data for genetic association with Type 2 diabetes (adjusted for BMI) was obtained from the DIAbetes Genetics Replication And Meta‐analysis (DIAGRAM) consortium comprised of 32 studies with 898 130 individuals of European descent [20]. The inverse‐variance weighted (IVW) method was used to estimate causal association between each rhythmic metabolite and Type 2 diabetes. Covariates in each cohort included in the MR analyses are presented in Table S2.

## 3 Results

At baseline, almost half of the participants were males, 42.0% were aged 65 years or older, and 52.8% had a bachelor's or higher education (Table 1). The prevalence of Type 2 diabetes at baseline was 10.6% and the incidence of Type 2 diabetes at the 3‐year follow‐up was 3.4%.

**TABLE 1:** Descriptive characteristics of study participants at baseline (*n* = 9992).

	*n*	%
Age (years)		
45–54	2521	25.2
55–64	3274	32.8
65–74	2443	24.4
≥ 75	1754	17.6
Sex		
Male	4906	49.1
Female	5086	50.9
Education status		
No postsecondary education	739	8.7
Diploma or certificate below bachelor's	3249	38.5
Bachelor's degree	2308	27.3
Above bachelor's degree	2150	25.5
Annual household income (CAD)		
< $50 000	2650	28.3
$50 000 to < $100 000	3308	35.3
$100 000 to < $150 000	1885	20.1
≥ $150 000	1530	16.3
Adequate physical activity	3086	32.3
Cigarette smoking		
Non‐smoker or former smoker	5914	86.9
Occasional smoker (≥ 1 cigarette in lifetime)	168	2.5
Current daily smoker	724	10.6
Alcohol consumption		
Never	1159	11.9
Daily or occasional	8597	88.1
BMI (kg/m^2^) (mean, SD)	28.1	5.4
Diet quality score (Mean, SD)	14.0	4.9
Use of lipid lowering medication	2812	28.1
Number of chronic conditions		
None	1643	16.4
One	2107	21.1
Two or more	6242	62.5

### 3.1 Association Between Chronotype PRS and Rhythmic Metabolites

**FIGURE 1:**
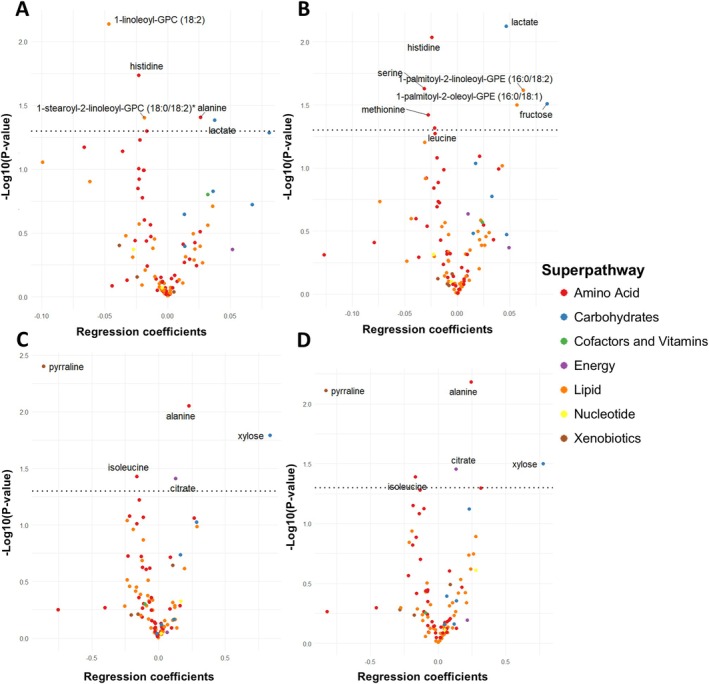
Volcano plot showing the regression coefficient and −log10 (*p* value) for polygenic risk score (A: morningness based on all risk alleles; B: morning person based on all risk alleles; C: morningness based on core clock genes (*CLOCK*, *BMAL1*, *PER1*, *PER2*, *PER3*, *CRY1*, *CRY2*, *MTNR1B*) and D: Morning person based on core clock genes) with rhythmic metabolites. All models were adjusted for age, sex, smoking, adequate physical activity, satisfaction with current sleep pattern, sleep duration, stop breathing in sleep, participation in shift work, number of chronic conditions and 20 genetic ancestry principal components. Black dotted line denotes statistical significance threshold for false discovery rate (FDR)‐adjusted *p* value < 0.05. Positive regression coefficient indicates positive association with morningness chronotype traits.

Figure 1 illustrates the association between PRS for chronotype traits and rhythmic metabolites across four models. For the morningness trait, significant metabolites included alanine, histidine, 1‐linoleoyl‐GPC (18:2), 1‐stearoyl‐2‐linoleoyl‐GPC (18:0/18:2), and lactate (Panel A). For the morning person trait, significant metabolites included histidine, leucine, serine, methionine, 1‐palmitoyl‐2‐linoleoyl‐GPE (16:0/18:2), 1‐palmitoyl‐2‐oleoyl‐GPE (16:0/18:1), lactate, and fructose (Panel B). For both, morningness and morning‐person traits based on core clock genes, significant metabolites included pyrraline, isoleucine, alanine, citrate, and xylose (Panel C and Panel D).

### 3.2 Association Between Rhythmic Metabolites and Type 2 Diabetes Risk

The associations between metabolites previously identified as rhythmic and incident Type 2 diabetes risk after adjusting for covariates are presented in Table S4. Of the 98 metabolites included, significant associations with Type 2 diabetes were observed for 20 metabolites (20.4%), including 10 amino acids, 6 lipids, 3 carbohydrates and 1 nucleotide. All these metabolites were also associated with Type 2 diabetes risk in the testing dataset. Of them, a subset of metabolites (isoleucine, 1‐linoleoyl‐GPC (18:2), 1‐palmitoyl‐2‐linoleoyl‐GPE (16:0/18:2) and 1‐palmitoyl‐2‐oleoyl‐GPE (16:0/18:1), and lactate) were also associated with PRS for chronotype. This overlap provides evidence that the selected rhythmic metabolites are not only rhythmic but also partly influenced by chronotype. Furthermore, their association with both Type 2 diabetes and chronotype PRS supports the potential role of circadian metabolic processes in Type 2 diabetes pathophysiology.

#### 3.2.1 Amino Acid Metabolism

Within the leucine, isoleucine, and valine metabolism sub‐pathway, branch‐chain amino acids (BCAAs), including valine (RR = 1.35; 95% CI = 1.09–1.67) and isoleucine (RR = 1.23; 95% CI = 1.02–1.48), were associated with higher Type 2 diabetes risk. Moreover, their metabolic derivatives, 3‐methyl‐2‐oxovalerate (RR = 1.23; 95% CI = 1.06–1.42) and 4‐methyl‐2‐oxopentanoate (RR = 1.18; 95% CI = 1.02–1.37), were also positively associated with Type 2 diabetes risk. Other metabolites, including glutamate (RR = 1.31; 95% CI = 1.20–1.44), 5‐hydroxylysine (RR = 1.08; 95% CI = 1.00–1.17) and creatine (RR = 1.11; 95% CI = 1.01–1.21), were positively associated with Type 2 diabetes risk. Conversely, three metabolites within the glycine, serine, and threonine metabolism sub‐pathway, including threonine (RR = 0.80; 95% CI = 0.66–0.97), glycine (RR = 0.66; 95% CI = 0.54–0.79) and betaine (RR = 0.83; 95% CI = 0.69–0.99), were inversely associated with Type 2 diabetes risk.

#### 3.2.2 Lipid Metabolism

Three metabolites related to phosphatidylethanolamine metabolism sub‐pathway, including 1‐palmitoyl‐2‐oleoyl‐GPE (16:0/18:1) (RR = 1.21; 95% CI = 1.10–1.32), 1‐palmitoyl‐2‐linoleoyl‐GPE (16:0/18:2) (RR = 1.16; 95% CI = 1.06–1.28), and 1‐stearoyl‐2‐oleoyl‐GPE (18:0/18:1) (RR = 1.18; 95% CI = 1.08–1.28) were positively associated with Type 2 diabetes risk. Further, sphingomyelin (d18:0/18:0, d19:0/17:0) (RR = 1.14; 95% CI = 1.04–1.25) was positively associated with Type 2 diabetes risk. Metabolites within the lysophospholipid metabolism sub‐pathway, including higher levels of 1‐oleoyl‐GPC (18:1) (RR = 0.79; 95% CI = 0.67–0.94) and 1‐linoleoyl‐GPC (18:2) (RR = 0.83; 95% CI = 0.73–0.94) were associated with a lower Type 2 diabetes risk.

#### 3.2.3 Carbohydrate Metabolism

As expected, glucose (RR = 1.85; 95% CI = 1.40–2.46), mannose (RR = 1.64; 95% CI = 1.39–1.94) and lactate (RR = 1.14; 95% CI = 1.01–1.30) were associated with higher Type 2 diabetes risk.

#### 3.2.4 Nucleotide Metabolism

Xanthine (RR = 1.11; 95% CI = 1.04–1.17), a product of purine metabolism and a precursor of uric acid, was associated with higher Type 2 diabetes risk.

### 3.3 Metabolic Pathway Enrichment Analysis

**FIGURE 2:**
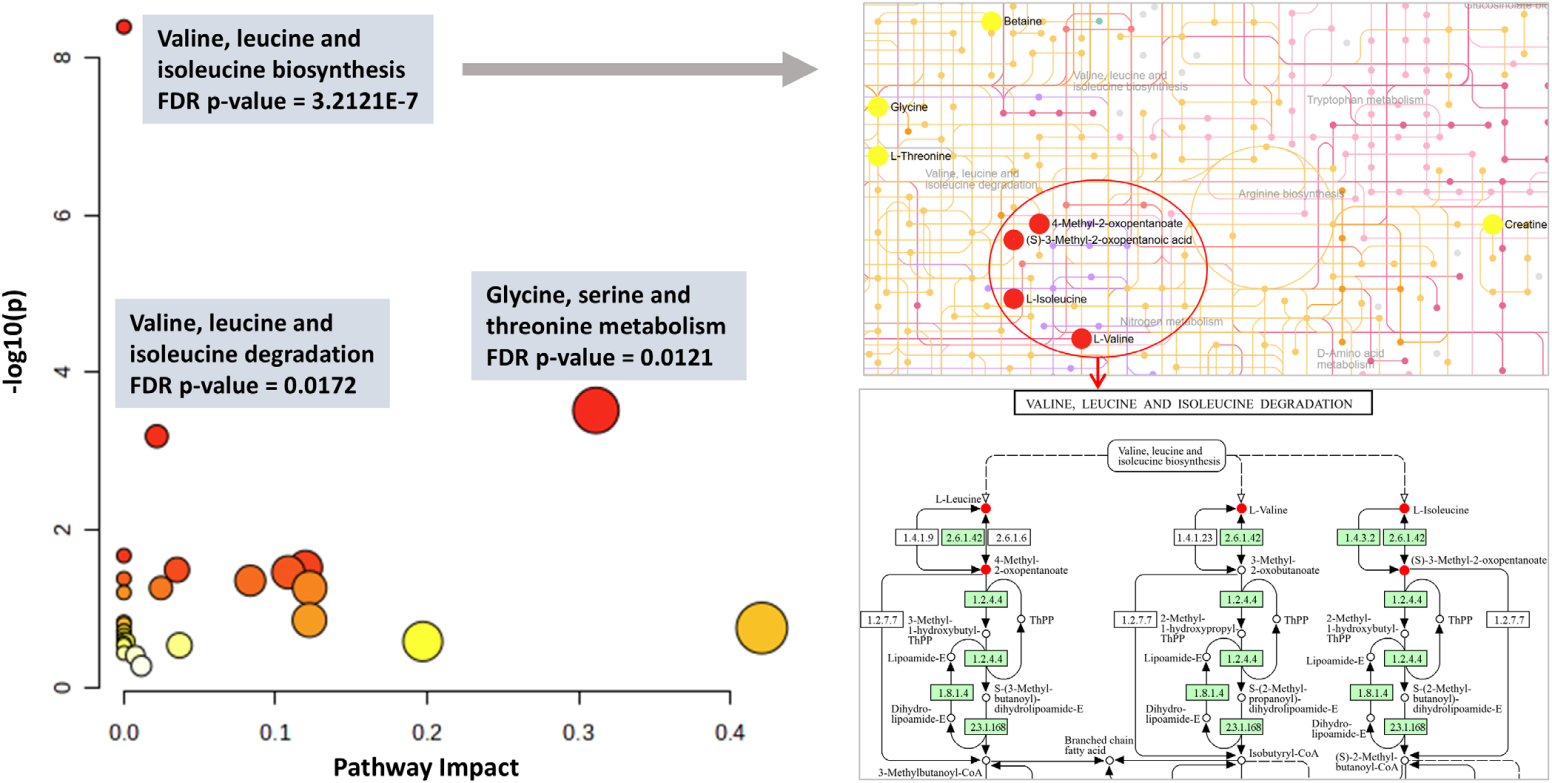
Pathway analysis based on enrichment analysis identifying the most relevant metabolic pathways. KEGG pathway enrichment analysis of rhythmic metabolites significantly associated with incident Type 2 diabetes. Only metabolites that were both significantly associated and mapped to KEGG pathways are shown. Other metabolites were assessed in the study but were either not significantly associated or could not be mapped to KEGG pathways. A full list of all metabolites and their association estimates is provided in Table S4.

We performed KEGG pathway enrichment analysis of Type 2 diabetes associated metabolites to understand their biological significance (Figure 2). Figure 2 displays only the subset of rhythmic metabolites that were both significantly associated with Type 2 diabetes and could be mapped to KEGG pathways. Other metabolites included in the study were either not significantly associated with Type 2 diabetes or could not be mapped to KEGG pathways. A complete list of all metabolites and their association estimates is provided in Table S4. The analysis showed three significant enrichment pathways including valine, leucine, and isoleucine biosynthesis (FDR *p* value = 3.21E‐7, Figure S1) and degradation (FDR *p* value = 0.0172, Figure S2), and glycine, serine and threonine metabolism (FDR *p* value = 0.0121, Figure S3).

### 3.4 Mendelian Randomisation Analysis

**FIGURE 3:**
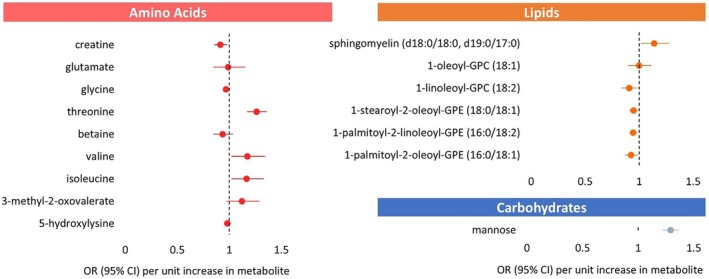
Forest plot of MR‐analysed blood metabolites on Type 2 diabetes causality. Mendelian randomisation estimates and *p* values were calculated using an inverse‐variance weighted fixed effects model for instruments that contained more than one variant and a Wald ratio test for instruments with one variant.

Of the 20 rhythmic metabolites that were associated with Type 2 diabetes in observational analysis, genetic instruments were identified for 16 metabolites based on data from metabolomics GWAS. Two‐sample MR was conducted on these 16 metabolites, of which 11 metabolites showed evidence supporting a potential causal association with Type 2 diabetes (Figure 3). Suitable SNPs were not available for glucose, lactate, 4‐methyl‐2‐oxopentanoate and xanthine, and were therefore excluded from MR analysis. The results showed higher genetically predicted levels of mannose (OR = 1.29; 95% CI = 1.22–1.37), BCAAs valine (OR = 1.17; 95% CI = 1.02–1.34) and isoleucine (OR = 1.17; 95% CI = 1.02–1.33), and sphingomyelin (d18:0/18:0, d19:0/17:0) (OR = 1.14; 95% CI = 1.02–1.28) were associated with genetically predicted higher Type 2 diabetes risk, whereas glycine (OR = 0.97; 95% CI = 0.94–0.99) and 1‐linoleoyl‐GPC (18:2) (OR = 0.91; 95% CI = 0.84–0.99) were associated with genetically predicted lower Type 2 diabetes risk. The direction of effect for a few metabolites differed from that observed in the observational analyses, consistent with a potential causal role. Creatine (OR = 0.91; 95% CI = 0.85–0.98) and 1‐palmitoyl‐2‐oleoyl‐GPE (16:0/18:1) (OR = 0.92; 95% CI = 0.87–0.99), 1‐palmitoyl‐2‐linoleoyl‐GPE (16:0/18:2) (OR = 0.94; 95% CI = 0.91–0.98), and 1‐stearoyl‐2‐oleoyl‐GPE (18:0/18:1) (OR = 0.95; 95% CI = 0.90–0.99) were associated with a genetically predicted lower risk, whereas threonine (OR = 1.26; 95% CI = 1.17–1.36) was associated with a higher Type 2 diabetes risk.

## 4 Discussion

We found 20 metabolites to be associated with incident Type 2 diabetes at 3 years. Although associations between individual metabolites and Type 2 diabetes have been reported previously, our study focused specifically on metabolites consistently identified as rhythmic in prior research. A subset of these metabolites also overlapped with genetic predisposition to chronotype, reinforcing that circadian processes may contribute to Type 2 diabetes risk. By capturing the broader set of rhythmic metabolites, while emphasising those linked to chronotype, we provide a comprehensive and mechanistically informative assessment of circadian metabolic contributions to Type 2 diabetes. Many of the associated metabolites were amino acids and lipids involved in biologically important pathways, including BCAA and glycine, serine and threonine metabolism. MR analysis provided evidence for a causal relationship between higher levels of mannose, valine, isoleucine and sphingomyelin (d18:0/18:0, d19:0/17:0) and higher Type 2 diabetes risk, while higher levels of glycine and 1‐linoleoyl‐GPC (18:2) were associated with lower Type 2 diabetes risk. The direction of causal effect for some metabolites including creatine, threonine, and phosphatidylethanolamines differed from observational associations. These findings suggest that metabolites under circadian regulation may contribute to Type 2 diabetes pathogenesis through altered levels or regulation.

Circadian rhythms play an important role in maintaining metabolic processes, including regulation of BCAA catabolism. Circadian rhythm genes *CLOCK*, *PER3* and *BMAL1* regulate the expression of *KLF15*, which in turn regulates BCAA catabolism gene *BCAT2* [21, 22]. Therefore, disruptions in circadian rhythm due to biological or environmental factors can lead to the dysregulation (reduction) of *KLF15*. This, in turn, promotes the expression of *BCAT2*, altering the BCAA catabolic pathway and ultimately resulting in elevated levels of BCAAs [21]. BCAAs have been shown to be rhythmic in the skeletal muscle of humans [23]. Our results suggest that an imbalance in BCAAs can impair glucose homeostasis and insulin sensitivity, which is observed in individuals with Type 2 diabetes [24]. Chronotype PRS was associated with circulating BCAAs, suggesting that inherited circadian factors may contribute alongside behavioural and environmental factors to amino acid regulation. MR findings are also consistent with a potential contribution of these BCAAs to Type 2 diabetes risk.

The Glycine, Serine, and Threonine Metabolism pathway was significantly enriched in our pathway analysis, highlighting its importance in Type 2 diabetes pathogenesis. Lower betaine levels are associated with decreased S‐adenosylmethionine, which in turn may decrease phosphatidylcholine synthesis, increase hepatic fat buildup, and alter very low‐density lipoprotein synthesis and secretion [25]. Low betaine levels are also associated with high triglycerides and phospholipid transfer protein activity [26]. Thus, betaine deficiency may increase Type 2 diabetes risk through disruption of lipid metabolism. Betaine metabolism also contributes to the formation of glycine [27, 28]. L‐glycine supplementation has been shown to improve sleep quality and reduce daytime sleepiness and fatigue, possibly through the activation of N‐methyl‐D‐aspartate receptors in the suprachiasmatic nucleus [29, 30]. Our findings support these results that suggest that glycine supplementation could offer potential intervention strategies for reducing Type 2 diabetes risk by mitigating the effects of circadian disruption.

The contrasting findings for threonine in MR analysis support the diverse role of functional amino acids in metabolic regulation. Dietary threonine supplementation has been shown to improve insulin resistance in mice studies and lipid metabolism in human studies [31, 32]. However, genetically predicted higher threonine levels were associated with Type 2 diabetes, consistent with a potential causal influence, suggesting the possibility of disrupted lipid metabolism involvement in Type 2 diabetes pathogenesis. Previous research has shown threonine dehydrogenase, an enzyme involved in threonine catabolism, is inhibited by certain fatty acids and their derivatives [33]. Thus, elevated lipid levels may impair threonine breakdown, resulting in higher circulating levels.

Our results showed that genetically predicted elevated creatine levels were associated with lower Type 2 diabetes risk, consistent with a potential causal role, which contrasts with findings from our observational analysis. Other observational studies have found higher plasma creatine levels to be associated with Type 2 diabetes [34], whereas in experimental trials, creatine supplementation has been shown to improve glucose metabolism in healthy and insulin‐resistant individuals [35]. Recent research suggests that elevated circulating creatine levels in Type 2 diabetes may be a consequence rather than the cause of impaired creatine metabolism [36].

Taken together, these findings highlight that some metabolites, including creatine and threonine, do not conform to expected patterns based solely on their presumed circadian rhythmicity or MR‐predicted causal effects. Based on current evidence, we hypothesise that elevated circulating creatine in observational analyses may reflect compensatory metabolic adaptations and impaired skeletal muscle mitochondrial function, potentially related to reduced mitochondrial creatine kinase 2 (*CKMT2*) expression, rather than a direct causal role in Type 2 diabetes [36]. Meanwhile, threonine levels may be modulated by interactions with lipid metabolism, as an experimental study showed that threonine dehydrogenase activity is inhibited by fatty acids and ketone‐related metabolites [33], suggesting that altered threonine levels arise secondary to lipid‐driven mitochondrial dysfunction. Future mechanistic studies in cell or animal models, as well as longitudinal or repeated‐measures metabolomics in humans, could test these hypotheses, disentangle direct circadian regulation from secondary metabolic effects, and clarify their role in Type 2 diabetes risk.

Several phosphatidylethanolamines and sphingomyelin (d18:0/18:0, d19:0/17:0) were associated with higher risk, whereas most phosphatidylcholines were associated with lower Type 2 diabetes risk in our observational analyses. Some of these findings were further supported by MR, which identified a potential causal role for sphingomyelin in increasing Type 2 diabetes risk, and 1‐linoleoyl‐GPC (18:2) in decreasing Type 2 diabetes risk, with the latter also associated with PRS for chronotype. Our findings are consistent with research indicating reduced lysophospholipid levels in individuals with Type 2 diabetes [29, 30]. For phosphatidylethanolamines, the fatty acid chain length and saturation can influence Type 2 diabetes risk through their effects on membrane fluidity, signalling and mitochondrial function [37]. We observed phosphatidylethanolamines containing saturated fatty acids to be associated with higher Type 2 diabetes risk. Phosphatidylethanolamines are highly enriched in mitochondria and the inner leaflet of the plasma membrane, where they play a critical role in lipid homeostasis, glucose metabolism and systemic energy regulation [6], and phosphatidylethanolamine metabolism dysregulation is implicated in Type 2 diabetes development [38]. Thus, lipid metabolites that exhibit circadian or diurnal oscillations may link circadian regulation with Type 2 diabetes risk. Interestingly, phosphatidylethanolamines were associated with a lower Type 2 diabetes risk in MR. Supporting this, evidence from in vivo and in vitro models showed decreased levels of phosphatidylethanolamines due to downregulation of phosphoethanolamine cytidylyltransferase in hepatocytes of Type 2 diabetes mice [39]. However, this specific mechanism needs to be confirmed in future studies.

Although we selected metabolites previously reported as rhythmic in at least three independent studies, only a subset showed significant associations with PRS for chronotype. This is not unexpected, as prior reports of rhythmic metabolites arise from diverse experimental contexts, sampling protocols, and analytical platforms, and may capture behavioural rather than genetically driven rhythms. Consequently, our findings suggest that only a subset of metabolites exhibit variation directly influenced by inherited circadian mechanisms. Future studies will be essential to delineate which rhythmic metabolites reflect direct circadian gene regulation and which are driven by secondary behavioural or environmental influences.

## 5 Strengths and Limitations

The sample was predominantly of European ancestry, which may limit the generalizability of the findings to more diverse populations. A key limitation of this study is that metabolomics data were collected at a single time point, precluding direct assessment of circadian rhythmicity within the CLSA. Accordingly, rhythmic metabolite classification relied on consistent evidence from prior studies. While the study included 70% of the metabolites previously identified as exhibiting rhythmicity, some relevant metabolites were not included due to lack of available data. The 3‐year follow‐up period may not be sufficient to fully assess the onset and long‐term risk of developing Type 2 diabetes. Although we adjusted for several known risk factors, residual confounding remains possible, including from variables related to chronotype. Chronotype‐related variability may reflect both genetic circadian regulation and behavioural factors such as sleep timing, light exposure, and lifestyle. In our analysis of PRS and rhythmic metabolites, we adjusted for relevant covariates including shift work, diet quality, BMI, and physical activity; however, it is not possible to fully separate genetic and behavioural influences due to lack of direct measures of sleep and other circadian behaviours. In the analysis of rhythmic metabolites and Type 2 diabetes, residual behavioural influences related to chronotype could also contribute to observed associations. Some rhythmic metabolites may also partly reflect dietary patterns or glucose metabolism rather than intrinsic circadian regulation, but the results remained consistent after accounting for diet quality, BMI, and fasting status. Further, pathway analyses do not capture tissue‐specific uptake, release, or metabolism, and cannot reliably identify which tissue(s) contribute to the observed circulating metabolite changes. Also, pathway analysis could not be conducted for a few metabolites as they could not be mapped in KEGG, thereby potentially leading to the loss of information on pathways relevant to T2D risk. Lastly, the use of serum samples for metabolite assessment may not fully reflect metabolic processes occurring at the tissue or organ level, potentially limiting mechanistic interpretation. Nevertheless, the CLSA is a well‐characterised, nationally generalizable cohort, with metabolomics data available for nearly 10 000 individuals, which allowed for robust testing of the associations. Type 2 diabetes was comprehensively assessed, minimising misclassification bias, and MR analysis strengthened causal inferences between rhythmic metabolites and Type 2 diabetes.

## 6 Implications and Conclusions

Overall, these results suggest that disruptions in circadian metabolic regulation may contribute to Type 2 diabetes development through specific metabolite pathways and reinforce growing evidence that maintaining circadian alignment is essential for metabolic health. Our findings provide evidence for developing metabolite‐based biomarkers and targeted interventions to improve circadian and metabolic health. Public health strategies aimed at reducing chronic circadian disruptions, such as promoting regular sleep–wake patterns, aligning meal timing with natural light–dark cycles, minimising exposure to artificial light at night, and mitigating the metabolic impacts of shift work, could support metabolic regulation and complement other clinical and health behaviour strategies to reduce Type 2 diabetes risk. Future studies should evaluate whether modifying circadian behaviours or targeting these metabolic pathways can effectively prevent or delay the onset of Type 2 diabetes.

## Author Contributions

D.J., T.R., M.P. and P.R. were involved in the conception, design, and conduct of the study. D.J. wrote the first draft of the manuscript. D.J., T.R., M.P., R.d.M., F.R., D.C., J.‐P.D., A.C.C., J.H., P.S. and P.R. were involved in the analysis and interpretation of the results. All authors edited, reviewed and approved the final version of the manuscript. D.J. and P.R. are the guarantor of this work and, as such, had full access to all the data in the study and take responsibility for the integrity of the data and the accuracy of the data analysis.

## Funding

This study was funded by The Netherlands Organization for Health Research and Development (ZonMw) (459001021), Dutch Diabetes Research Foundation (Diabetes Fonds) (2019.11.101), the Canadian Institutes of Health Research (CIHR) (TNC‐174963), and Health‐Holland (LSHM20107). This collaborative project is co‐financed with a PPP‐allowance made available by Health‐Holland, Topsector Life Sciences & Health, to stimulate public‐private partnerships. The study sponsor/funder was not involved in the design of the study; the collection, analysis, and interpretation of data; writing the report; and did not impose any restrictions regarding the publication of the report.

## Disclosure

The opinions expressed in this manuscript are the author's own and do not reflect the views of the Canadian Longitudinal Study on Aging.

## Conflicts of Interest

The authors declare no conflicts of interest.

## Supporting information


**Figure S1:** Valine, leucine and isoleucine biosynthesis pathway.
**Figure S2:** Valine, leucine and isoleucine degradation pathway.
**Figure S3:** Glycine, serine and threonine metabolism pathway.
**Table S1:** Plasma metabolites associated with circadian rhythm identified in three or more studies were available in the CLSA.
**Table S2:** Covariates adjusted in external GWAS used for calculation of polygenic risk score and two‐sample Mendelian randomisation analyses.
**Table S3:** Covariates adjusted for in the models examining the association between metabolites and Type 2 diabetes risk in the CLSA.
**Table S4:** Association of circadian rhythm metabolites with incidence Type 2 diabetes.

## Data Availability

Data are available from the Canadian Longitudinal Study on Aging (www.clsa‐elcv.ca) for researchers who meet the criteria for access to de‐identified CLSA data.
