# Supplementary material for: Circulating Rhythmic Metabolites and Causal Risk of Type 2 Diabetes in Adults in the Canadian Longitudinal Study on Aging
Source: Diabetes Obes Metab. 2026 Mar 8;28(5):4243–52. doi: 10.1111/dom.70616 (PMC13071190; doi:10.1111/dom.70616)
Supplement: Supplementary file 1 — Figure S1: Valine, leucine and isoleucine biosynthesis pathway. Figure S2: Valine, leucine and isoleucine degradation pathway. Figure S3: Glycine, serine and threonine metabolism pathway. Table S1: Plasma metabolites associated with circadian rhythm identified in three or more studies were available in the CLSA. Table S2: Covariates adjusted in external GWAS used for calculation of polygenic risk score and two‐sample Mendelian randomisation analyses. Table S3: Covariates adjusted for in the models examining the association between metabolites and Type 2 diabetes risk in the CLSA. Table S4: Association of circadian rhythm metabolites with incidence Type 2 diabetes. [file DOM-28-4243-s001.docx]

**Circulating rhythmic metabolites and causal risk of type 2 diabetes in adults in the Canadian Longitudinal Study on Aging**

Divya Joshi, Talha Rafiq, Marie Pigeyre, Renee de Mutsert, Femke Rutters, David Campbell, Jean-Pierre Despres, Andre C. Carpentier, Joris Hoeks, Patrick Schrauwen, Parminder Raina

**Supplementary Material**

Table of Contents

[Methods 3](#_Toc214271857)

[Figures 5](#_Toc214271858)

[Tables 8](#_Toc214271859)

# Methods

**Metabolomics**

Each sample extract was separated into four aliquots and processed for analysis in the positive, negative, and polar ion mode. Metabolites were identified by automated comparison of ion features to the proprietary reference library of chemical standard entries that contains the retention time/index, mass to charge ratio (m/z), and chromatographic data (including MS/MS spectral data). A series of quality control and curation procedures were conducted for accurate and consistent identification of chemical entities and to remove entities representing system artifacts, misassignments, and background noise [1]. Data are available for a total of 1,314 metabolites from a wide range of biochemical classes including amino acids, carbohydrates, lipids, xenobiotics, choline, polyamine, cofactor, and vitamins. A data-normalization method was performed by Metabolon according to their standard protocols to correct variations resulting from instrument inter-day tuning differences and to account for potential batch effects [1].

**Polygenic risk score for chronotype**

Briefly, phasing and imputation were conducted using the TOPMed reference panel at the University of Michigan Imputation Service. We used the TOPMed reference panel version r2, and then pre-phased and imputed the genotype data using EAGLE2 and Minimac, respectively, for both autosomal and X chromosomes. Samples with low call rates (<95%), sex mismatches, or cryptic relatedness were removed. Imputed SNPs were excluded based on low call rates (<95%), deviation from Hardy-Weinberg (p<10-6), low minor allele frequency (MAF<0.0001), and low imputation quality (Rsq < 0.6). We constructed polygenic risk scores (PRS) for chronotype using previously identified genetic variants to investigate whether the rhythmic metabolites were associated with genetic predisposition to chronotype as we did not have questionnaire data on chronotype.

PRS were computed using LASSOSUM, a penalized regression-based method that enables fine-tuning of parameters [2]. Specifically, LASSOSUM applies penalization to GWAS effect sizes, optimizing the selection of variants and shrinkage of effect sizes based on a reference linkage disequilibrium panel. Variants were filtered using a significance threshold of P<0.01 to prioritize genetic variants with stronger evidence of association. PRS calculations were performed for each trait by summing the weighted effect sizes of the selected variants, where weights were derived from the penalized regression model.

# Figures


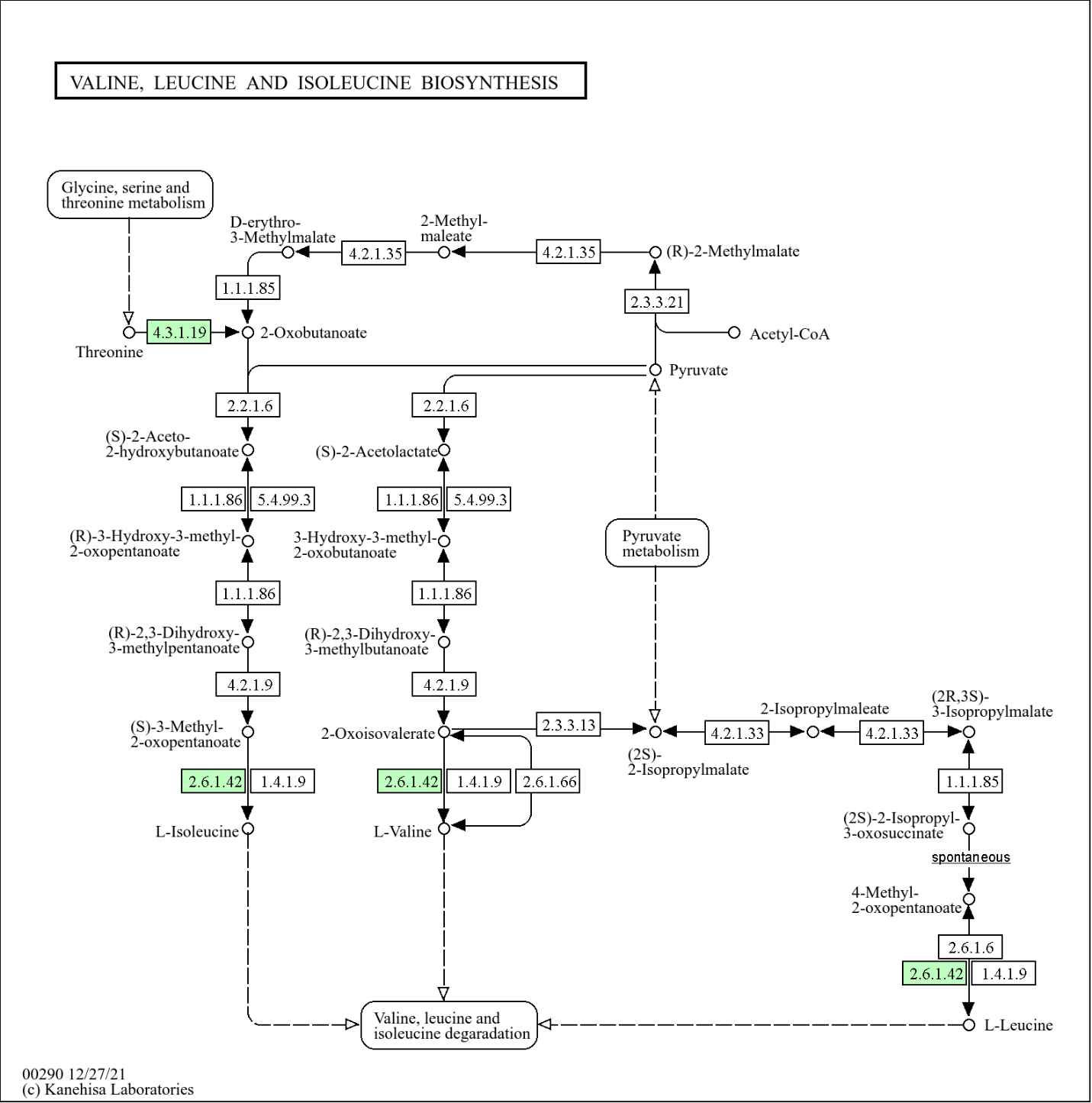


**Figure S1 – Valine, Leucine, and Isoleucine Biosynthesis Pathway**


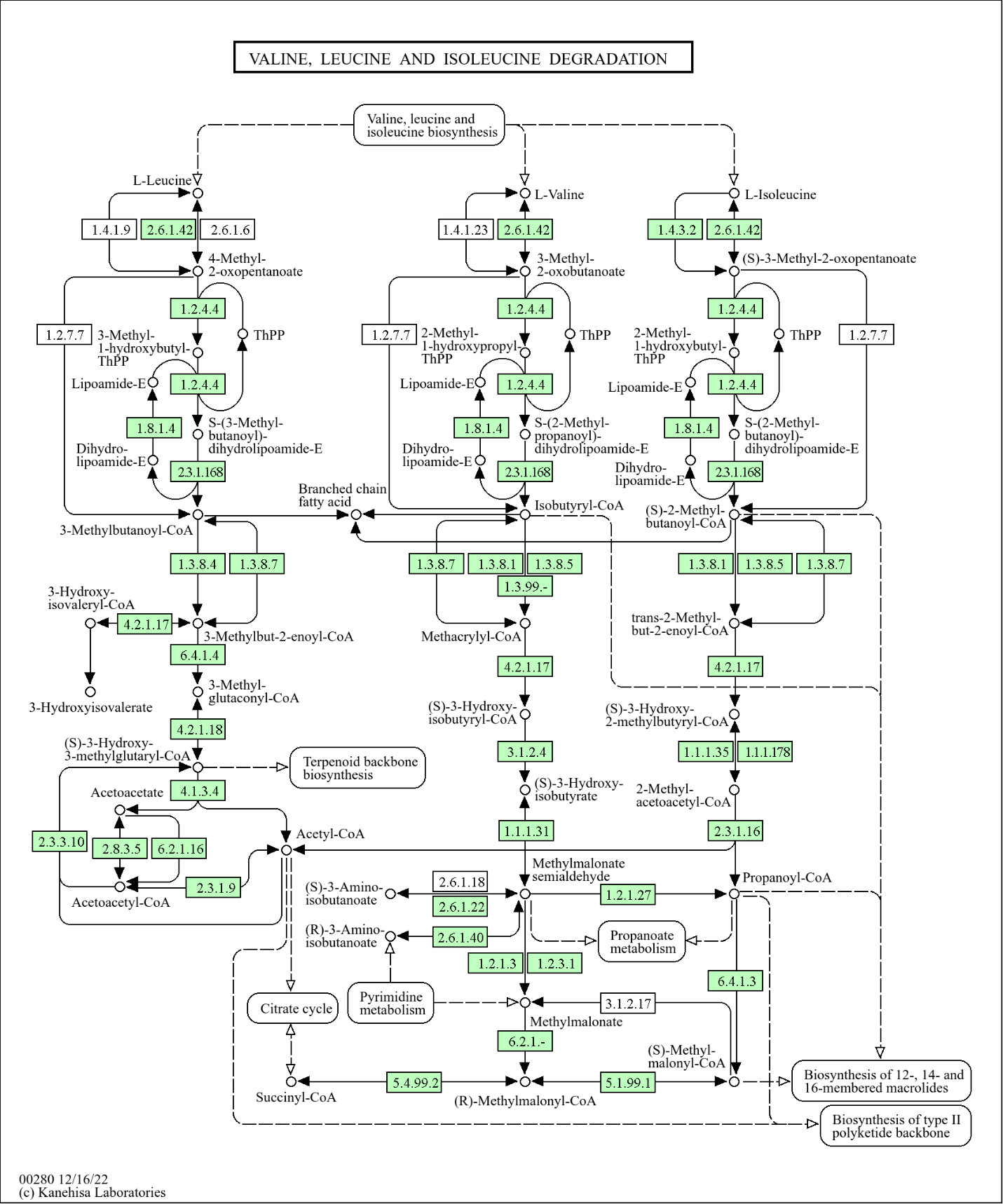


**Figure S2 – Valine, Leucine, and Isoleucine Degradation Pathway**


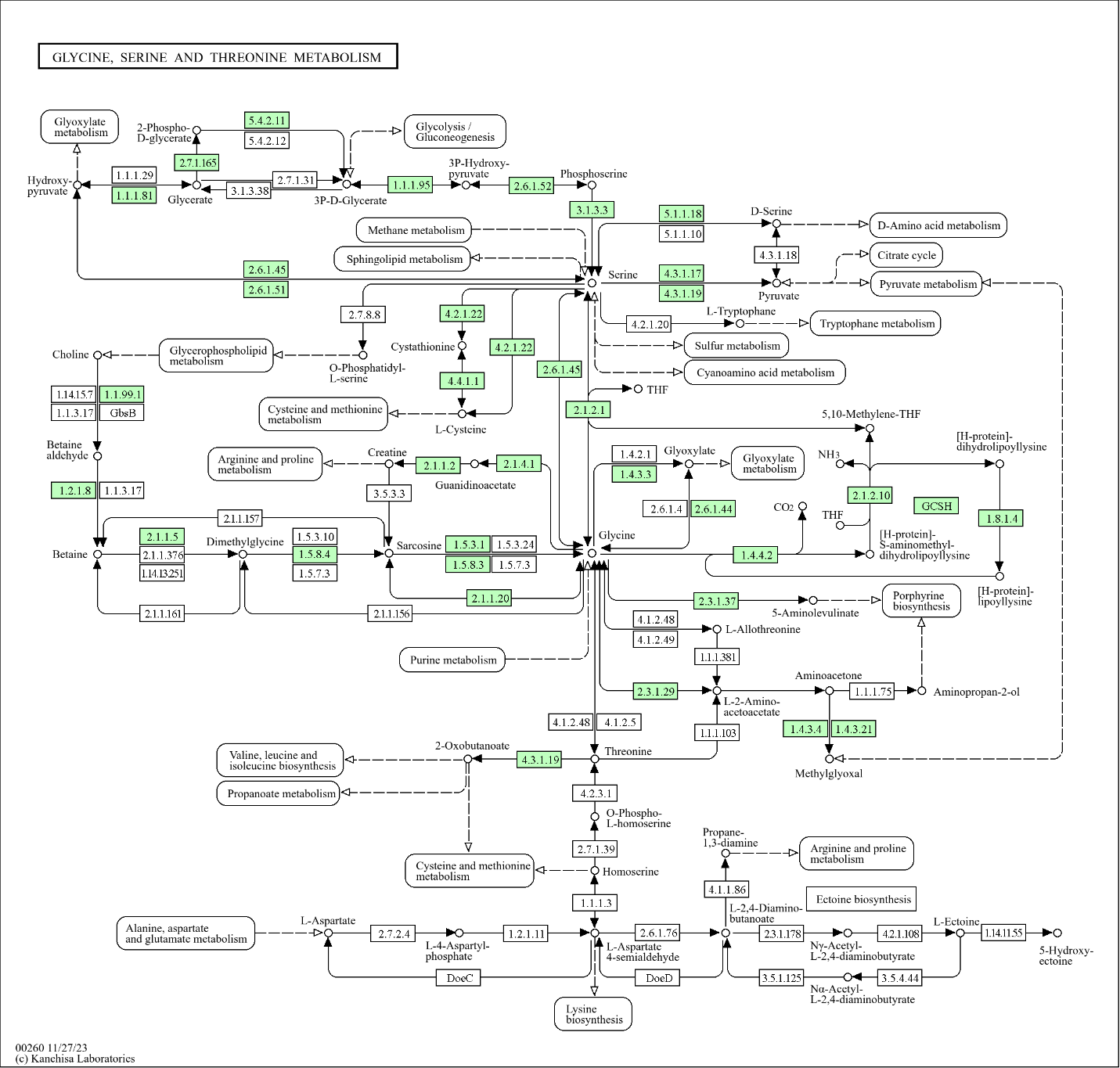


**Figure S3 – Glycine, Serine and Threonine Metabolism Pathway**

# Tables

**Table S1: Plasma metabolites associated with circadian rhythm identified in 3 or more studies were available in the CLSA***

| **Metabolite** | **Subpathway** | **Superpathway** | **Frequency** |
| --- | --- | --- | --- |
| proline | Urea cycle; Arginine and Proline Metabolism | Amino Acid | 11 |
| leucine | Leucine, Isoleucine and Valine Metabolism | Amino Acid | 10 |
| 1,2-dipalmitoyl-GPC (16:0/16:0) | Phosphatidylcholine (PC) | Lipid | 10 |
| phenylalanine | Phenylalanine Metabolism | Amino Acid | 9 |
| tyrosine | Tyrosine Metabolism | Amino Acid | 9 |
| ornithine | Urea cycle; Arginine and Proline Metabolism | Amino Acid | 9 |
| glutamate | Glutamate Metabolism | Amino Acid | 8 |
| isoleucine | Leucine, Isoleucine and Valine Metabolism | Amino Acid | 8 |
| 1-linoleoyl-GPC (18:2) | Lysophospholipid | Lipid | 8 |
| valine | Leucine, Isoleucine and Valine Metabolism | Amino Acid | 7 |
| taurine | Methionine, Cysteine, SAM and Taurine Metabolism | Amino Acid | 7 |
| tryptophan | Tryptophan Metabolism | Amino Acid | 7 |
| citrulline | Urea cycle; Arginine and Proline Metabolism | Amino Acid | 7 |
| cortisone | Corticosteroids | Lipid | 6 |
| creatinine | Creatine Metabolism | Amino Acid | 6 |
| glycine | Glycine, Serine and Threonine Metabolism | Amino Acid | 6 |
| 1-oleoyl-GPC (18:1) | Lysophospholipid | Lipid | 6 |
| 1-palmitoyl-GPC (16:0) | Lysophospholipid | Lipid | 6 |
| citrate | TCA Cycle | Energy | 6 |
| kynurenine | Tryptophan Metabolism | Amino Acid | 6 |
| alanine | Alanine and Aspartate Metabolism | Amino Acid | 5 |
| cortisol | Corticosteroids | Lipid | 5 |
| sarcosine | Glycine, Serine and Threonine Metabolism | Amino Acid | 5 |
| threonine | Glycine, Serine and Threonine Metabolism | Amino Acid | 5 |
| lysine | Lysine Metabolism | Amino Acid | 5 |
| trimethylamine N-oxide | Phospholipid Metabolism | Lipid | 5 |
| pregnenolone sulfate | Pregnenolone Steroids | Lipid | 5 |
| carnitine | Carnitine Metabolism | Lipid | 4 |
| creatine | Creatine Metabolism | Amino Acid | 4 |
| acetylcarnitine (C2) | Fatty Acid Metabolism (Acyl Carnitine, Short Chain) | Lipid | 4 |
| glutamine | Glutamate Metabolism | Amino Acid | 4 |
| glucose | Glycolysis, Gluconeogenesis, and Pyruvate Metabolism | Carbohydrate | 4 |
| lactate | Glycolysis, Gluconeogenesis, and Pyruvate Metabolism | Carbohydrate | 4 |
| bilirubin (Z,Z) | Hemoglobin and Porphyrin Metabolism | Cofactors and Vitamins | 4 |
| 1-methylhistidine | Histidine Metabolism | Amino Acid | 4 |
| histidine | Histidine Metabolism | Amino Acid | 4 |
| 3-hydroxyisobutyrate | Leucine, Isoleucine and Valine Metabolism | Amino Acid | 4 |
| 3-methyl-2-oxovalerate | Leucine, Isoleucine and Valine Metabolism | Amino Acid | 4 |
| alpha-hydroxyisocaproate | Leucine, Isoleucine and Valine Metabolism | Amino Acid | 4 |
| 1-stearoyl-GPC (18:0) | Lysophospholipid | Lipid | 4 |
| 1-myristoyl-2-palmitoyl-GPC (14:0/16:0) | Phosphatidylcholine (PC) | Lipid | 4 |
| xanthine | Purine Metabolism, (Hypo)Xanthine/Inosine containing | Nucleotide | 4 |
| 4-hydroxyphenylpyruvate | Tyrosine Metabolism | Amino Acid | 4 |
| arginine | Urea cycle; Arginine and Proline Metabolism | Amino Acid | 4 |
| urea | Urea cycle; Arginine and Proline Metabolism | Amino Acid | 4 |
| asparagine | Alanine and Aspartate Metabolism | Amino Acid | 3 |
| hippurate | Benzoate Metabolism | Xenobiotics | 3 |
| sulfate* | Chemical | Xenobiotics | 3 |
| N-palmitoyl-sphinganine (d18:0/16:0) | Dihydroceramides | Lipid | 3 |
| sphingomyelin (d18:0/18:0, d19:0/17:0) | Dihydrosphingomyelins | Lipid | 3 |
| oleoylcarnitine (C18:1) | Fatty Acid Metabolism (Acyl Carnitine, Monounsaturated) | Lipid | 3 |
| propionylcarnitine (C3) | Fatty Acid Metabolism (also BCAA Metabolism) | Lipid | 3 |
| (16 or 17)-methylstearate (a19:0 or i19:0) | Fatty Acid, Branched | Lipid | 3 |
| 2,3-dihydroxyisovalerate | Food Component/Plant | Xenobiotics | 3 |
| pyrraline | Food Component/Plant | Xenobiotics | 3 |
| fructose | Fructose, Mannose and Galactose Metabolism | Carbohydrate | 3 |
| mannose | Fructose, Mannose and Galactose Metabolism | Carbohydrate | 3 |
| glycerol | Glycerolipid Metabolism | Lipid | 3 |
| betaine | Glycine, Serine and Threonine Metabolism | Amino Acid | 3 |
| serine | Glycine, Serine and Threonine Metabolism | Amino Acid | 3 |
| 4-guanidinobutanoate | Guanidino and Acetamido Metabolism | Amino Acid | 3 |
| 3-hydroxybutyrate (BHBA) | Ketone Bodies | Lipid | 3 |
| 4-methyl-2-oxopentanoate | Leucine, Isoleucine and Valine Metabolism | Amino Acid | 3 |
| 10-heptadecenoate (17:1n7) | Long Chain Monounsaturated Fatty Acid | Lipid | 3 |
| myristoleate (14:1n5) | Long Chain Monounsaturated Fatty Acid | Lipid | 3 |
| oleate/vaccenate (18:1) | Long Chain Monounsaturated Fatty Acid | Lipid | 3 |
| dihomo-linoleate (20:2n6) | Long Chain Polyunsaturated Fatty Acid (n3 and n6) | Lipid | 3 |
| dihomo-linolenate (20:3n3 or n6) | Long Chain Polyunsaturated Fatty Acid (n3 and n6) | Lipid | 3 |
| docosadienoate (22:2n6) | Long Chain Polyunsaturated Fatty Acid (n3 and n6) | Lipid | 3 |
| docosapentaenoate (n6 DPA; 22:5n6) | Long Chain Polyunsaturated Fatty Acid (n3 and n6) | Lipid | 3 |
| linolenate [alpha or gamma; (18:3n3 or 6)] | Long Chain Polyunsaturated Fatty Acid (n3 and n6) | Lipid | 3 |
| nonadecanoate (19:0) | Long Chain Saturated Fatty Acid | Lipid | 3 |
| 5-hydroxylysine | Lysine Metabolism | Amino Acid | 3 |
| N6-acetyllysine | Lysine Metabolism | Amino Acid | 3 |
| 1-arachidonoyl-GPC (20:4n6) | Lysophospholipid | Lipid | 3 |
| 1-palmitoleoyl-GPC (16:1) | Lysophospholipid | Lipid | 3 |
| 1-stearoyl-GPE (18:0) | Lysophospholipid | Lipid | 3 |
| methionine | Methionine, Cysteine, SAM and Taurine Metabolism | Amino Acid | 3 |
| methionine sulfoxide | Methionine, Cysteine, SAM and Taurine Metabolism | Amino Acid | 3 |
| N-acetylmethionine | Methionine, Cysteine, SAM and Taurine Metabolism | Amino Acid | 3 |
| arabonate/xylonate | Pentose Metabolism | Carbohydrate | 3 |
| xylose | Pentose Metabolism | Carbohydrate | 3 |
| 1-linoleoyl-2-arachidonoyl-GPC (18:2/20:4n6) | Phosphatidylcholine (PC) | Lipid | 3 |
| 1-myristoyl-2-arachidonoyl-GPC (14:0/20:4) | Phosphatidylcholine (PC) | Lipid | 3 |
| 1-stearoyl-2-arachidonoyl-GPC (18:0/20:4) | Phosphatidylcholine (PC) | Lipid | 3 |
| 1-stearoyl-2-linoleoyl-GPC (18:0/18:2) | Phosphatidylcholine (PC) | Lipid | 3 |
| 1-oleoyl-2-linoleoyl-GPE (18:1/18:2) | Phosphatidylethanolamine (PE) | Lipid | 3 |
| 1-palmitoyl-2-linoleoyl-GPE (16:0/18:2) | Phosphatidylethanolamine (PE) | Lipid | 3 |
| 1-palmitoyl-2-oleoyl-GPE (16:0/18:1) | Phosphatidylethanolamine (PE) | Lipid | 3 |
| 1-stearoyl-2-oleoyl-GPE (18:0/18:1) | Phosphatidylethanolamine (PE) | Lipid | 3 |
| choline | Phospholipid Metabolism | Lipid | 3 |
| acisoga | Polyamine Metabolism | Amino Acid | 3 |
| spermidine | Polyamine Metabolism | Amino Acid | 3 |
| uridine | Pyrimidine Metabolism, Uracil containing | Nucleotide | 3 |
| sphingomyelin (d18:1/18:1, d18:2/18:0) | Sphingomyelins | Lipid | 3 |
| succinate | TCA Cycle | Energy | 3 |
| dimethylarginine (SDMA + ADMA) | Urea cycle; Arginine and Proline Metabolism | Amino Acid | 3 |
| N-acetylarginine | Urea cycle; Arginine and Proline Metabolism | Amino Acid | 3 |

*47% were amino acids, 42% were lipids, 6% were carbohydrates, 4% were xenobiotics, 2% each were energy and nucleotides, and 1% was cofactor and vitamins

**Table S2: Covariates adjusted in external GWAS used for calculation of polygenic risk score and two-sample Mendelian randomization analyses**

| **Variable** |
| --- |
| **UK Biobank (PRS for chronotype)** [3] |
| Age (years) |
| Sex (male, female) |
| Study centre |
| Genotyping array/release |
| Population structure |
| **CLSA (genetic variants for metabolites)** [4] |
| Age (years) |
| Sex (male, female) |
| Hour since last meal or drink |
| Genotyping batch |
| First ten genetic principal components |
| **Five studies: ARIC, FHS, CHS, MESA, and HCHS/SOL (genetic variants for metabolites)** [5] |
| Age (years) |
| Sex (male, female) |
| Race |
| Study |
| Study variables (such as recruitment center) |
| First 11 principal components with random effects accounting for inter-individual correlation (due to relatedness, shared household, or census block group) |
| **32 Studies (genetic variants for diabetes)** [6] |
| Age (years) |
| Sex (male, female) |
| Population stratification (using genetic principal components) |
| Body mass index (BMI) |

**Table S3: Covariates adjusted for in the models examining the association between metabolites and type 2 diabetes risk in the CLSA**

| **Variable** | **Measured/Operationalized** |
| --- | --- |
| Age | 45-54, 55-64, 65-74, ≥75 years |
| Sex | Male, Female |
| Education status | Less than high school, high school, below bachelor’s, bachelor’s or above |
| Smoking status | Former or never, occasional, current daily smoker |
| Physical activity | Adequate physical activity was assessed using the Physical Activity Scale for the Elderly (PASE), which measures the frequency, duration, and intensity of participation in leisure, household, and occupational or volunteer activities [7]. Participants were classified as participating in ‘adequate’ or ‘inadequate’ levels of physical activity based on the World Health Organization’s age-specific guideline of at least 75 minutes of vigorous-intensity or 150 minutes of moderate-intensity physical activity per week [8]. |
| Diet quality score | Dietary intake was assessed using the CLSA Short Diet Questionnaire, a validated tool designed to estimate usual food consumption over the past 12 months, particularly for an aging population [9]. Following the Prospective Urban Rural Epidemiological (PURE) healthy diet score framework, participants were categorized into quintiles of usual intake for each of the seven food groups, including fruits, vegetables, nuts, legumes, fish, dairy, and meats. Quintile values were then summed to generate an overall diet score ranging from 0 to 28, where higher scores reflect a poorer diet quality. |
| Alcohol consumption | Never, occasional or daily drinker |
| Body mass index | Kg/m^2^ |
| Shift work status | Shift work was assessed based on participants' work schedule in the job they worked the longest over their lifetime, classifying them as having participated in shift work or not. |
| Number of chronic conditions | Chronic conditions from ten disease categories including musculoskeletal, respiratory, cardiovascular, endocrine-metabolic, neurological, gastrointestinal, genitourinary, ophthalmologic, renal, and cancer were assessed via self-report with participants reporting only conditions diagnosed by a healthcare professional and lasting or expected to last at least six months. Each condition was assessed as present or absent, summed into a total score, and categorized as ‘none’, ‘one’, or ‘two or more’ for analysis. |
| Use of lipid modifying medications | Determined based on ATC code starting with C10 |

**Table S4: Association of circadian rhythm metabolites with incidence type 2 diabetes***

| **Metabolite** | **RR** | **95% CI** | **Super Pathway** | **Sub Pathway** |
| --- | --- | --- | --- | --- |
| 3-methyl-2-oxovalerate | 1.23 | 1.06, 1.42 | Amino Acid | Leucine, Isoleucine and Valine Metabolism |
| methionine sulfoxide | 1.00 | 0.89, 1.12 | Amino Acid | Methionine, Cysteine, SAM and Taurine Metabolism |
| 5-hydroxylysine | 1.08 | 1.00, 1.17 | Amino Acid | Lysine Metabolism |
| 4-guanidinobutanoate | 0.99 | 0.93, 1.05 | Amino Acid | Guanidino and Acetamido Metabolism |
| kynurenine | 1.02 | 0.98, 1.06 | Amino Acid | Tryptophan Metabolism |
| 4-methyl-2-oxopentanoate | 1.18 | 1.02, 1.37 | Amino Acid | Leucine, Isoleucine and Valine Metabolism |
| alpha-hydroxyisocaproate | 1.01 | 0.90, 1.14 | Amino Acid | Leucine, Isoleucine and Valine Metabolism |
| 1-methylhistidine | 1.07 | 0.96, 1.20 | Amino Acid | Histidine Metabolism |
| N-acetylarginine | 1.03 | 0.92, 1.15 | Amino Acid | Urea cycle; Arginine and Proline Metabolism |
| N6-acetyllysine | 0.96 | 0.80, 1.17 | Amino Acid | Lysine Metabolism |
| dimethylarginine (SDMA + ADMA) | 0.90 | 0.69, 1.17 | Amino Acid | Urea cycle; Arginine and Proline Metabolism |
| acisoga | 1.07 | 0.97, 1.17 | Amino Acid | Polyamine Metabolism |
| sarcosine | 0.99 | 0.88, 1.12 | Amino Acid | Glycine, Serine and Threonine Metabolism |
| N-acetylmethionine | 0.99 | 0.87, 1.12 | Amino Acid | Methionine, Cysteine, SAM and Taurine Metabolism |
| 3-hydroxyisobutyrate | 1.02 | 0.93, 1.10 | Amino Acid | Leucine, Isoleucine and Valine Metabolism |
| 4-hydroxyphenylpyruvate | 1.05 | 0.98, 1.12 | Amino Acid | Tyrosine Metabolism |
| creatine | 1.11 | 1.01, 1.21 | Amino Acid | Creatine Metabolism |
| arginine | 1.03 | 0.95, 1.12 | Amino Acid | Urea cycle; Arginine and Proline Metabolism |
| creatinine | 0.77 | 0.56, 1.06 | Amino Acid | Creatine Metabolism |
| glycine | 0.66 | 0.54, 0.79 | Amino Acid | Glycine, Serine and Threonine Metabolism |
| histidine | 1.00 | 0.76, 1.32 | Amino Acid | Histidine Metabolism |
| isoleucine | 1.23 | 1.02, 1.48 | Amino Acid | Leucine, Isoleucine and Valine Metabolism |
| citrulline | 1.07 | 0.87, 1.32 | Amino Acid | Urea cycle; Arginine and Proline Metabolism |
| leucine | 1.20 | 0.96, 1.50 | Amino Acid | Leucine, Isoleucine and Valine Metabolism |
| lysine | 1.09 | 0.84, 1.40 | Amino Acid | Lysine Metabolism |
| methionine | 1.04 | 0.88, 1.22 | Amino Acid | Methionine, Cysteine, SAM and Taurine Metabolism |
| ornithine | 0.97 | 0.85, 1.10 | Amino Acid | Urea cycle; Arginine and Proline Metabolism |
| phenylalanine | 1.23 | 0.94, 1.61 | Amino Acid | Phenylalanine Metabolism |
| proline | 1.01 | 0.85, 1.18 | Amino Acid | Urea cycle; Arginine and Proline Metabolism |
| spermidine | 0.95 | 0.87, 1.03 | Amino Acid | Polyamine Metabolism |
| serine | 0.90 | 0.75, 1.09 | Amino Acid | Glycine, Serine and Threonine Metabolism |
| taurine | 0.83 | 0.68, 1.01 | Amino Acid | Methionine, Cysteine, SAM and Taurine Metabolism |
| urea | 1.07 | 0.94, 1.21 | Amino Acid | Urea cycle; Arginine and Proline Metabolism |
| glutamate | 1.31 | 1.20, 1.44 | Amino Acid | Glutamate Metabolism |
| glutamine | 1.00 | 0.99, 1.02 | Amino Acid | Glutamate Metabolism |
| threonine | 0.80 | 0.66, 0.97 | Amino Acid | Glycine, Serine and Threonine Metabolism |
| tryptophan | 1.12 | 0.91, 1.39 | Amino Acid | Tryptophan Metabolism |
| valine | 1.35 | 1.09, 1.67 | Amino Acid | Leucine, Isoleucine and Valine Metabolism |
| betaine | 0.83 | 0.69, 0.99 | Amino Acid | Glycine, Serine and Threonine Metabolism |
| alanine | 1.06 | 0.87, 1.30 | Amino Acid | Alanine and Aspartate Metabolism |
| tyrosine | 1.14 | 0.96, 1.34 | Amino Acid | Tyrosine Metabolism |
| asparagine | 1.01 | 0.98, 1.03 | Amino Acid | Alanine and Aspartate Metabolism |
| arabonate/xylonate | 0.99 | 0.89, 1.09 | Carbohydrate | Pentose Metabolism |
| lactate | 1.14 | 1.01, 1.30 | Carbohydrate | Glycolysis, Gluconeogenesis, and Pyruvate Metabolism |
| glucose | 1.85 | 1.40, 2.46 | Carbohydrate | Glycolysis, Gluconeogenesis, and Pyruvate Metabolism |
| mannose | 1.64 | 1.39, 1.94 | Carbohydrate | Fructose, Mannose and Galactose Metabolism |
| xylose | 1.00 | 0.95, 1.05 | Carbohydrate | Pentose Metabolism |
| fructose | 1.01 | 0.96, 1.06 | Carbohydrate | Fructose, Mannose and Galactose Metabolism |
| bilirubin (Z,Z) | 0.95 | 0.85, 1.06 | Cofactors and Vitamins | Hemoglobin and Porphyrin Metabolism |
| citrate | 1.09 | 0.86, 1.38 | Energy | TCA Cycle |
| succinate | 0.99 | 0.95, 1.02 | Energy | TCA Cycle |
| carnitine | 1.05 | 0.83, 1.33 | Lipid | Carnitine Metabolism |
| 1,2-dipalmitoyl-GPC (16:0/16:0) | 0.86 | 0.67, 1.11 | Lipid | Phosphatidylcholine (PC) |
| 1-myristoyl-2-palmitoyl-GPC (14:0/16:0) | 1.07 | 0.97, 1.17 | Lipid | Phosphatidylcholine (PC) |
| acetylcarnitine (C2) | 1.00 | 0.89, 1.13 | Lipid | Fatty Acid Metabolism (Acyl Carnitine, Short Chain) |
| propionylcarnitine (C3) | 1.07 | 0.96, 1.19 | Lipid | Fatty Acid Metabolism (also BCAA Metabolism) |
| docosadienoate (22:2n6) | 1.06 | 0.99, 1.14 | Lipid | Long Chain Polyunsaturated Fatty Acid (n3 and n6) |
| myristoleate (14:1n5) | 1.02 | 0.96, 1.08 | Lipid | Long Chain Monounsaturated Fatty Acid |
| 1-palmitoyl-GPC (16:0) | 0.84 | 0.67, 1.06 | Lipid | Lysophospholipid |
| 1-stearoyl-GPC (18:0) | 0.86 | 0.73, 1.01 | Lipid | Lysophospholipid |
| 1-oleoyl-GPC (18:1) | 0.79 | 0.67, 0.94 | Lipid | Lysophospholipid |
| 10-heptadecenoate (17:1n7) | 1.05 | 0.99, 1.11 | Lipid | Long Chain Monounsaturated Fatty Acid |
| linolenate [alpha or gamma; (18:3n3 or 6)] | 1.03 | 0.97, 1.09 | Lipid | Long Chain Polyunsaturated Fatty Acid (n3 and n6) |
| 1-linoleoyl-GPC (18:2) | 0.83 | 0.73, 0.94 | Lipid | Lysophospholipid |
| 1-stearoyl-GPE (18:0) | 0.98 | 0.84, 1.14 | Lipid | Lysophospholipid |
| oleoylcarnitine (C18:1) | 1.01 | 0.90, 1.15 | Lipid | Fatty Acid Metabolism (Acyl Carnitine, Monounsaturated) |
| 1-palmitoleoyl-GPC (16:1)* | 0.99 | 0.87, 1.13 | Lipid | Lysophospholipid |
| 1-arachidonoyl-GPC (20:4n6)* | 0.89 | 0.79, 1.00 | Lipid | Lysophospholipid |
| docosapentaenoate (n6 DPA; 22:5n6) | 1.03 | 0.96, 1.10 | Lipid | Long Chain Polyunsaturated Fatty Acid (n3 and n6) |
| dihomo-linolenate (20:3n3 or n6) | 1.07 | 0.98, 1.15 | Lipid | Long Chain Polyunsaturated Fatty Acid (n3 and n6) |
| 1-stearoyl-2-oleoyl-GPE (18:0/18:1) | 1.18 | 1.08, 1.28 | Lipid | Phosphatidylethanolamine (PE) |
| 1-stearoyl-2-arachidonoyl-GPC (18:0/20:4) | 1.07 | 0.85, 1.36 | Lipid | Phosphatidylcholine (PC) |
| 1-palmitoyl-2-linoleoyl-GPE (16:0/18:2) | 1.16 | 1.06, 1.28 | Lipid | Phosphatidylethanolamine (PE) |
| sphingomyelin (d18:1/18:1, d18:2/18:0) | 0.95 | 0.78, 1.17 | Lipid | Sphingomyelins |
| pregnenolone sulfate | 0.98 | 0.93, 1.03 | Lipid | Pregnenolone Steroids |
| (16 or 17)-methylstearate (a19:0 or i19:0) | 1.03 | 0.96, 1.10 | Lipid | Fatty Acid, Branched |
| trimethylamine N-oxide | 1.07 | 0.99, 1.15 | Lipid | Phospholipid Metabolism |
| oleate/vaccenate (18:1) | 1.07 | 0.99, 1.16 | Lipid | Long Chain Monounsaturated Fatty Acid |
| 1-stearoyl-2-linoleoyl-GPC (18:0/18:2)* | 1.07 | 0.79, 1.44 | Lipid | Phosphatidylcholine (PC) |
| sphingomyelin (d18:0/18:0, d19:0/17:0)* | 1.14 | 1.04, 1.25 | Lipid | Dihydrosphingomyelins |
| N-palmitoyl-sphinganine (d18:0/16:0) | 1.10 | 0.99, 1.22 | Lipid | Dihydroceramides |
| 1-oleoyl-2-linoleoyl-GPE (18:1/18:2)* | 1.07 | 0.99, 1.15 | Lipid | Phosphatidylethanolamine (PE) |
| 1-linoleoyl-2-arachidonoyl-GPC (18:2/20:4n6)* | 0.91 | 0.77, 1.09 | Lipid | Phosphatidylcholine (PC) |
| 1-myristoyl-2-arachidonoyl-GPC (14:0/20:4)* | 1.09 | 0.99, 1.20 | Lipid | Phosphatidylcholine (PC) |
| dihomo-linoleate (20:2n6) | 1.04 | 0.97, 1.11 | Lipid | Long Chain Polyunsaturated Fatty Acid (n3 and n6) |
| glycerol | 1.00 | 0.97, 1.03 | Lipid | Glycerolipid Metabolism |
| choline | 1.00 | 0.91, 1.10 | Lipid | Phospholipid Metabolism |
| 1-palmitoyl-2-oleoyl-GPE (16:0/18:1) | 1.21 | 1.10, 1.32 | Lipid | Phosphatidylethanolamine (PE) |
| 3-hydroxybutyrate (BHBA) | 1.00 | 0.96, 1.05 | Lipid | Ketone Bodies |
| cortisone | 1.04 | 0.93, 1.15 | Lipid | Corticosteroids |
| cortisol | 1.06 | 0.97, 1.15 | Lipid | Corticosteroids |
| nonadecanoate (19:0) | 1.02 | 0.90, 1.14 | Lipid | Long Chain Saturated Fatty Acid |
| xanthine | 1.11 | 1.04, 1.17 | Nucleotide | Purine Metabolism, (Hypo)Xanthine/Inosine containing |
| uridine | 1.09 | 0.99, 1.20 | Nucleotide | Pyrimidine Metabolism, Uracil containing |
| hippurate | 1.00 | 0.95, 1.05 | Xenobiotics | Benzoate Metabolism |
| pyrraline | 0.99 | 0.93, 1.04 | Xenobiotics | Food Component/Plant |
| 2,3-dihydroxyisovalerate | 0.98 | 0.94, 1.01 | Xenobiotics | Food Component/Plant |
| sulfate* | 1.13 | 0.95, 1.34 | Xenobiotics | Chemical |

*All models were adjusted for age, sex, education status, smoking, physical activity, alcohol consumption, diet quality, body mass index, number of chronic conditions, and use of lipid lowering medication

**References**

[1] Michelotti G, Wong K, Forgetta V, et al. (2023) Metabolomic Profiling on 9,992 Participants using Ultra-performance Liquid Chromatography and Mass Spectrometer. CLSA Data Support Document. Available from <https://www.clsa-elcv.ca/wp-content/uploads/2024/01/CLSA_DataSupportDoc_Metabolomics_v2.0_2023Aug03.pdf>

[2] Choi SW, Mak TS, O'Reilly PF (2020) Tutorial: a guide to performing polygenic risk score analyses. Nature protocols 15(9): 2759-2772. 10.1038/s41596-020-0353-1

[3] Jones SE, Lane JM, Wood AR, et al. (2019) Genome-wide association analyses of chronotype in 697,828 individuals provides insights into circadian rhythms. Nature Communications 10(1): 343. <http://dx.doi.org/10.1038/s41467-018-08259-7>

[4] Chen Y, Lu T, Pettersson-Kymmer U, et al. (2023) Genomic atlas of the plasma metabolome prioritizes metabolites implicated in human diseases. Nat Genet 55(1): 44-53. 10.1038/s41588-022-01270-1

[5] Feofanova EV, Brown MR, Alkis T, et al. (2023) Whole-Genome Sequencing Analysis of Human Metabolome in Multi-Ethnic Populations. Nat Commun 14(1): 3111. 10.1038/s41467-023-38800-2

[6] Mahajan A, Taliun D, Thurner M, et al. (2018) Fine-mapping type 2 diabetes loci to single-variant resolution using high-density imputation and islet-specific epigenome maps. Nat Genet 50(11): 1505-1513. 10.1038/s41588-018-0241-6

[7] Washburn RA, McAuley E, Katula J, Mihalko SL, Boileau RA (1999) The physical activity scale for the elderly (PASE): evidence for validity. J Clin Epidemiol 52(7): 643-651. 10.1016/s0895-4356(99)00049-9

[8] World Health Organization (2010) Global recommendations on physical activity for health [Internet]. World Health Organization, Geneva, Switzerland

[9] Shatenstein B, Payette H (2015) Evaluation of the Relative Validity of the Short Diet Questionnaire for Assessing Usual Consumption Frequencies of Selected Nutrients and Foods. Nutrients 7(8): 6362-6374. 10.3390/nu7085282
